# Significance of positive semi-quantitative PCR tests on bronchoalveolar lavage for *Pneumocystis jirovecii* pneumonia in HIV-negative immunocompromised ICU patients with acute respiratory failure

**DOI:** 10.1186/s13613-025-01568-3

**Published:** 2025-10-27

**Authors:** Louis-Maxime Vaconsin, Christine Bonnal, Nicolas Argy, Julien Dessajan, Paul-Henri Wicky, Michael Thy, Etienne de Montmollin, Romain Sonneville, Lila Bouadma, Sandrine Houzé, Jean-François Timsit

**Affiliations:** 1 Medical and Infectious Diseases ICU, Paris Cité University- Bichat University Hospital, Assistance Publique - Hôpitaux de Paris, Paris, France; 2 Department of Parasitology-Mycology, Paris Cité University- Bichat University Hospital, Assistance Publique - Hôpitaux de Paris, Paris, France; 3 Inserm UMR 1137 - IAME Team 3 - MOCLID INSERM/ université Paris-Cité, Paris, France

## Abstract

**Context:**

Real-time PCR (rt-PCR) using cycle threshold (Ct) is a semi-quantitative way to assess DNA amounts, which has become broadly used to diagnose *Pneumocystis jirovecii* pneumonia (PJP) in non-HIV immunocompromised patients. We aimed to describe the non-HIV immunocompromised patients hospitalized in intensive care unit (ICU) for acute respiratory failure (ARF) and to evaluate the relevance of PJP rt-PCR Ct value in diagnosing PJP. Moreover, the added value of serum 1.3 ß-D-glucan (BDG) assay in this population was also assessed.

**Methods:**

All non-HIV immunocompromised ICU patients with ARF with at least one rt-PCR performed in broncho-alveolar lavage (BAL) from 2013 to 2023 were retrospectively included. Patients with a positive RT-PCR were classified by reviewers aware of the PCR result, but blinded to Ct values, into confirmed, uncertain, or ruled-out PJP groups based on clinical presentation, imaging findings, organism identification, laboratory results, presence of alternative diagnoses, and the resolution of acute respiratory failure with or without appropriate PJP treatment. PJ rt-PCR Ct and BDG assays of each group were compared. Uncertain diagnoses were excluded from the primary analysis and successively considered as confirmed PJP or ruled-out PJP in a secondary analysis. Using the area under the curve (AUC) of the receiver operating characteristics curves, the best threshold of Ct value was defined.

**Results:**

Out of the 481 non-HIV immunocompromised patients who underwent a PJ rt-PCR in BAL, 59 (12%) had a positive test. The results confirmed PJP for 23/59 (39%), ruled it out for 27/59 (46%), while it remained uncertain for 9/59 (15%). Rt-PCR sensitivity and specificity were respectively 100% (95% CI = [85.7–100%]) and 94% (95% CI = [91.4–95.8%]). Median Ct and BDG levels differed significantly between the confirmed, uncertain, and ruled-out groups at 25, 31, and 34 cycles; and 523, 78, and 32 pg/ml, respectively. The primary analysis identified the best Ct to categorize patients at 30, with an AUC of 0.931 (95% CI [0.850–1.0]), a sensitivity of 86% and a specificity of 89%.

**Conclusions:**

Semi-quantitative PJ PCR was accurate in diagnosing PJP in non-HIV ICU patients with acute respiratory failure (ARF), and a Ct at low cycle values was more frequent in confirmed PJP than in colonization. The optimal Ct threshold was 30. The BDG assay was especially valuable when high levels were reached.

**Supplementary Information:**

The online version contains supplementary material available at 10.1186/s13613-025-01568-3.

## Introduction

First described in premature and malnourished children after World War II [1, 2], then in hematologic patients [3] in the 1960s and 1970s, and in HIV patients in the 1980s and 1990s, *Pneumocystis jirovecii* (PJ) pneumonia (PJP) has now become more common in non-HIV patients due to both the widespread use of antiretroviral therapies (ART) and the increased use of immunosuppressive drugs and diseases [4]. The main identified risk factors are impaired T-cell immunity due to steroid use, stem cell transplant, solid organ transplant (SOT), and the lack of prophylaxis [5].

Acute respiratory failure (ARF) in intensive care units (ICU) accounts for up to one third of the ICU admissions of immunocompromised patients [5]. In non-HIV immunocompromised patients, the diagnosis of PJP is often more difficult due to a sudden onset of symptoms, and a more severe and rapid respiratory failure leading to a higher mortality rate than in HIV patients [4, 6]. Early diagnosis of PJP is therefore critical for optimal patient outcomes.

Since PJ cannot be cultured, laboratory tools rely on specific staining and/or direct or indirect immunofluorescence (IF) to identify the pathogen, either cysts or trophozoites, the transmissible and infectious forms of the organism, respectively. However, staining techniques are time-consuming and require trained technicians, and IF performance varies widely depending on the reagent used (sensitivity 48–100%, specificity 82–100%) [7]. Since PJ is an almost obligate alveolar pathogen with a decreasing gradient from the alveoli to the upper respiratory tract, bronchoalveolar lavage fluid (BALF) remains the gold standard specimen [7], although the technique is invasive and risky [8–10]. Notably, the fungal load is lower in non-HIV immunocompromised patients, and specific staining is therefore less sensitive [11].

In this regard, polymerase chain reaction (PCR) tests, which are more sensitive than direct examination and specific for PJ [12], have become a central component in the PJP diagnosis. Conventional or qualitative PCR tests (cPCR) are valuable for ruling out PJP because false negatives are rare, but they lack specificity to differentiate between infection and colonization, defined as a positive test without clinical signs. Colonization is common in non-HIV immunocompromised patients [13] and immunocompetent patients [14, 15].

Nowadays, researchers focus on either quantitative or semi quantitative DNA measures, using real-time PCR (rt-PCR) to establish thresholds to differentiate infection from colonization, with a possible grey zone in between, as more DNA was shown to be associated with PJP diagnosis [12, 16–19], while in colonized patients, amounts of DNA are usually lower [17, 20]. Quantitative PCR tests express results in copies of DNA per milliliter (mL) or in log values per mL, but they require trained technicians and take time. Semi-quantitative PCR tests use the number of cycles of DNA amplification as a surrogate for DNA quantity. They are sold in automatic kits that are faster and easier to use than quantitative PCR. Several studies [12, 19, 21], including a prospective study [18], have evaluated cut-off values to differentiate between colonization and infection, but no consensus has been reached in current recommendations [22].

Beta-D-Glucan (BDG) is a component of many fungi membranes, and its serum level has also been used to help diagnose PJP in HIV or hematologic patients. A meta-analysis showed a sensitivity and specificity of 94.8% and 86.3%, respectively, but patients with other fungal infections were excluded, which may have artificially inflated diagnostic parameters [23]. This assay could be useful in conjunction with rt-PCR [24].

However, as highlighted by recent reviews [25, 26], the diagnostic value of PJ PCR in non-HIV immunocompromised patients in the ICU has not been studied, and the added value of serum BDG assay in this population is insufficiently known.

This retrospective single-center study was conducted to evaluate the diagnostic value of a positive rt-PCR for PJP in non-HIV immunocompromised patients admitted for acute respiratory failure in the ICU and its threshold, as well as the additional value of serum BDG assay.

## Materials and methods

### Study setting and objectives

The study was conducted in a single intensive care unit (ICU) in Paris, specializing in infectious diseases. All medical records of patients with ARF from 2013 to 2023 who underwent PJP rt-PCR testing strictly on bronchoalveolar lavage (BAL), based on clinical suspicion of PJP were retrospectively reviewed. Suspicion of PJP was based on an integrated assessment of clinical symptoms, radiological findings, and underlying immunosuppressive conditions, as judged by the treating clinicians at the time of care.

Patients under the age of 18, who did not meet immunosuppression criteria, were HIV-positive, or were diagnosed with Sars-Cov2 infection, were excluded.

We aimed to describe non-HIV immunocompromised patients admitted to the intensive care unit (ICU) with acute respiratory failure (ARF) due to PJP, and to evaluate the relevance of cycle threshold (Ct) values in its diagnosis.

The primary objective was to determine the diagnostic threshold of rt-PCR for PJP in non-HIV immunocompromised ICU patients. The secondary objective was to evaluate the added value of serum BDG assays in this specific population.

The study was approved by the IRB of the French Society of Intensive Care Medicine (SRLF) (CE SRLF 24–049).

### Definition

#### Immunosuppression

Immunosuppression was defined as being a solid organ transplant (SOT) recipient (SOTR), having an autoimmune condition, having a solid tumor, having hematologic disorders including immune deficiencies, receiving systemic steroid therapy for more than three months or at high doses (> 0.5 mg/kg/j), receiving immunosuppressive drugs other than for SOT, or receiving systemic anticancer therapies.

#### BDG thresholds

BDG in serum was assayed using the Fungitell^®^ kit (Associates of Cape Cod Inc., Falmouth, MA, USA), following the manufacturer’s instructions. The recommended cutoff value of 80 pg/ml was used to define positivity. The test’s maximum value was 523 pg/ml. Dilutions to measure concentrations above the maximum value were not performed.

#### Rt-PCR: specimen collection and processing

Bronchoalveolar lavage fluid (BALF) was the only eligible respiratory specimen. BALF was aliquoted into two parts: one for microscopic examination and one for rt-PCR processing. Until 2020, the extraction of total nucleic acids was performed on 200 µL native respiratory specimens using the EZ1 Advanced XL Qiagen (France, Courtaboeuf). Since 2020, 1000 µL of native respiratory specimens were extracted using the NucliSens easyMAG platform (bioMérieux, Marcy l’Etoile, France) according to the manufacturer’s instructions. Extracts were stored at − 20 °C until processing.

Microscopic detection of *P. jirovecii* was performed on BAL fluid specimens using May-Grunwald -Giemsa staining (BAL MGG).

During the study period, three rt-PCR techniques targeting the mitochondrial large subunit rRNA (mtLSU) were successively used: from 2014 to 2016, Bio-Evolution *Pneumocystis jirovecii* real-time PCR (Bio-Evolution, France, Paris) qPCR); from 2016 to March 2020, Fast track Diagnostics (FTD) *Pneumocystis* PCR kit (Luxembourg) and, since March 2020: altona RealStar *Pneumocystis jirovecii* PCR kit 1.0 (Germany, Hamburg).

PJP rt-PCR tests with Ct values below 40 cycles were considered positive.

Notably, quantitative PJP PCR was not performed, as it was not available in our hospital laboratory.

#### Classification

As per European recommendations [27], classification of non-HIV immunocompromised patients with a positive PJ rt-PCR test was based on case history, clinical, biological, radiological criteria, and differential diagnoses. We added clinical response to treatment to benefit from the retrospective nature of this study and an uncertain group to account for diagnostic uncertainty. Reviewers were blinded to Ct values, stored separately, but had access to BDG results.

Authors categorized patients with a positive PJ rt-PCR test into three diagnostic groups.

- Confirmed PJP included:
  - Proven PJP, i.e., with detection of the organism microscopically in BAL fluid associated with compatible clinical and radiologic criteria;
  - Probable PJP, i.e., with amplification of PJ DNA by semi-quantitative real-time PCR in BAL fluid associated with compatible clinical and radiologic criteria, and no differential diagnosis explaining both clinical and radiologic criteria and/or resolution of the respiratory failure with treatment against PJP;
- Ruled-out PJP was defined in case of amplification of PJ DNA without compatible clinical and radiological criteria, clinical cure without PJP-directed treatment (up to 7 days of empirical treatment accepted), and no relapse of ARF ultimately confirmed or attributed to PJP.
- In doubtful situations, patients were categorized as having an “uncertain PJP” diagnosis.

Compatible radiological criteria were defined as the new onset of predominantly perihilar ground-glass opacities, which may evolve into consolidation over time, without associated pleural effusion.

### Data collection

For all non-HIV immunocompromised patients with ARF, key clinical characteristics, diagnoses, treatments and outcome data were retrospectively collected.

The following data from patients with a positive rt-PCR test were extracted from their records: results of direct BAL examination, number of PJ rt-PCR cycles in BAL, clinical characteristics including comorbidities, organ failure, procedure performed, length of stay, and outcome.

SAPS II score, Charlson comorbidity index (CCI), and SOFA scores were computed. All diagnostic exams for ARF were collected, including general laboratory data, BDG, bacteriological and virological results, BAL fluid characteristics, cardiac echography and chest computerized tomography (CT) scan or chest X-ray if CT was not available. Treatments targeting PJP, pneumonia, or other causes of ARF were also recorded. All patients were followed up until death or March 2024.

### Statistical analysis

Descriptive data were expressed as median and interquartile ranges (IQR).

Differences were calculated using the Chi-squared (X^2^) test for qualitative variables and the Mann-Whitney-U or Kruskal Wallis test for quantitative variables, as appropriate.

Statistical analyses were performed using DATATAB, Excel software, or SPSS Statistics, version 29 (SPSS Inc., Chicago, IL), and a p-value < 0.05 was considered statistically significant.

#### Comparison between the positive and negative PCR groups, and Rt-PCR test performance

A negative PJP PCR result did not automatically exclude the diagnosis of PJP. Data regarding their clinical course and any potential PJP treatment were obtained. Based on PCR results, we compared the positive and negative PCR groups. After implementing our classification as the gold standard to define true and false positives, we constructed a contingency table, excluding uncertain diagnoses. The diagnostic value of the PJP PCR test was assessed for sensitivity, specificity, positive predictive value (PPV), negative predictive value (NPV), positive likelihood ratio (PLR), negative likelihood ratio (NLR), and effectiveness. We also calculated the prevalence of PJP and positive PJP rt-PCR tests.

#### Comparison between the confirmed PJP group, the uncertain PJP group and the ruled-out PJP groups

PJ rt-PCR cycles in BALs and serum BDG levels were analyzed across groups using Kruskal Wallis and Chi-squared tests. Post-hoc comparisons were conducted using a Dunn-Bonferroni test.

#### ROC curves and best Ct threshold

ROC curves were used to calculate area under the curve (AUC) for PCR tests. The Youden index was used to identify the optimal thresholds.

The primary analysis compared the confirmed PJP group to the ruled-out PJP group.

As a secondary analysis and to address ambiguity in the classification of the ‘uncertain PJP” group, ROC curves, AUCs, and the Youden index were calculated by alternatively classifying uncertain cases as either “confirmed PJP” or “ruled-out PJP”.

## Results

From 2013 to 2023, 481 non-HIV immunocompromised patients with acute respiratory failure, suspected of PJP, had at least one PJ rt-PCR run on BAL, of which 59 (12%) tested positive (flow chart is found in Supplemental Fig. 1).

Out of the 59 patients with a positive PCR, we categorized 23 patients (39%) as having confirmed PJP, 27 (46%) as having ruled-out PJP and 9 (15%) were classified as uncertain.

### Comparison between the positive and negative PCR groups

None of the patients with a negative PCR developed PJP later on, with a median follow-up until either the end of the study or death of 293 days. The prevalence of positive PJ PCR was significantly higher in patients with autoimmune diseases (47% vs. 24%, *p* < 0.001) and in those receiving immunomodulatory therapy (36% vs. 12%, *p* < 0.001). Details comparing positive and negative rt-PCR groups are displayed in Supplemental Table 1.

### Diagnostic value of PJ rt-PCR

Based on our categorization and setting aside the uncertain PJP diagnoses, the prevalence of PJP in our population was 5%, PJ rt-PCR sensitivity and specificity were 100% (IC=[85.7%-100%]), and 94% (IC=[91.4% – 95.8%]) respectively. Positive and negative predictive values were 46% (IC=[33%-59.6%]) and 100% (IC = [99.1–100]), positive and negative likelihood ratio were 16 (IC=[11.7–25.4]) and 0 (IC=[0-0.12]), and rt-PCR diagnostic accuracy was 94% (IC=[91–96%]).

### Comparison between the confirmed PJP group, the uncertain PJP group and the ruled-out PJP group

The comparison in terms of clinical presentation, including retained diagnoses and biological parameters, is shown in Table 1. Importantly, steroid exposure, compatible radiographic patterns – defined as bilateral diffuse ground glass opacites on thoracic CT scan or interstitial infiltrates on chest X-ray – and the duration between symptoms and ICU admission were significantly different. Notably, although not statistically significant, the proportion of patients with septic shock was lower in the confirmed PJP group.

**Table 1:** Comparison between the confirmed, uncertain and ruled out groups

Positive PCR population characteristics	Confirmed PJP *n* = 23 (39%)	Uncertain PJP *n* = 9 (15%)		Ruled out PJP *n* = 27 (46%)		*P* values
PJP diagnostic tests						
Ct (median/Q1-Q3) ^◊^	25 (22–28)	31 (29–32)		34 (32–35)		< 0.001
Identification of pathogen (trophozoites or cysts) (n, %)	8 (35%)	0		0		0.001
Serum BDG (median/Q1-Q3) ^⊕ pg/mL^	523 (452–523)	78 (29–149)		32 (12–69)		0.001
Parameters at ICU admission						
Age in years (median/Q1-Q3)	65 (59–70)	64 (58–69)		65 (61–73)		0.842
Sex female (n, %)	9 (39%)	4 (44%)		8 (30%)		0.653
BMI (median/Q1-Q3) ^⊕^	23 (21–25)	20 (19–22)		26 (19–31)		0.229
Leucocytes (median/Q1-Q3) ^♠^	9 (4–12)	14 (11–16)		12 (5–18)		0.547
Lymphocytes (median/Q1-Q3) ^♥^	0.5 (0.3–0.8)	1 (0.5–1.2)		0.8 (0.5–1)		0.888
Neutrophils (median/Q1-Q3) ^♦^	8 (7–10)	13 (7–16)		10 (5–16)		0.585
LDH (median/Q1-Q3) •	644 (384–792)	564 (443–603)		407 (281–562)		0.17
PCT (median/Q1-Q3)^α^	0.82 (0.33–1.67)	0.28 (0.21–0.88)		0.95 (0.19–7.81)		0.537
CRP (median/Q1-Q3) ^β^	246 (212–290)	177 (160–270)		128 (69–282)		0.431
Scores at ICU admission						
SAPS 2 (median/Q1-Q3) ^◊^	34 (27–39)	36 (22–46)		39 (30–62)		0.39
CCI (median/Q1-Q3) ^◊^	4 (3–6)	4 (2–5)		4 (3–5)		0.737
SOFA (median/Q1-Q3) ^◊^	4 (2–7)	3 (2–7)		6 (4–8)		0.106
Immunosuppressive conditions						
Solid organ transplant (n, %)	6 (26.1%)	2 (22.2%)		4 (14.81%)		0.607
Auto immune disease (n, %)	11 (47.8%)	4 (44.4%)		13 (48.2%)		0.981
Solid tumour (n, %)	3 (13%)	2 (22.2)		5 (18.5%)		0.789
Systemic glucocorticoid therapy before PJP suspicion (n, %)	20 (87%)	7 (77.8%)		14 (51.9%)		0.023
Hematologic disorders (n, %)	0	1 (11%)		1 (0.04%)		0.293
Immunomodulatory drugs (but SOT) (n, %)	9 (39%)	4 (44%)		8 (30%)		0.653
Recent systemic cancer treatments (n, %)	2 (9%)	2 (22%)		6 (22%)		0.402
Clinical parameters						
Delay between symptoms and ICU in days (median/Q1-Q3) ^♣^	5 (4–8)	7 (6–10)		1 (0–2)		< 0.001
Thoracic imaging findings						
Xray or Ct scan pattern compatible (n, %) *	23 (100%)	8 (89%)		11 (41%)		< 0.001
Bilateral GGO on Ct scan (n, %)	21 (100%)	9 (100%)		22 (64%)		0.02
Alveolar condensation on Ct scan (n, %)	2 (10%)	3 (33%)		12 (55%)		0.08
Isolated alveolar condensations on Ct scan (n, %)	0 (0%)	0 (0%)		3 (14%)		0.107
Pleural effusion on Ct scan (n, %)	0 (0%)	2 (22%)		22 (68%)		< 0.01
ICU course and interventions						
Length Of Stay (median/Q1-Q3)	11 (6–25)	7 (6–10)		6 (3–16)		0.162
Mechanical ventilation (n, %)	15 (65%)	7 (77.8%)		19 (70.4%)		0.779
Septic shock at the time of PJP suspicion (%)	2 (8.7%)	2 (22.2%)		10 (37%)		0.063
Duration of PJP treatment in days (median/ Q1-Q3)	21 (20.5–21)	9 (6–16)		0 (0–3)		< 0.001
ICU survival (n, %)	16 (70%)	2 (22%)		20 (74%)		0.015
Survival after ICU discharge						
1-year survival (n, %)	12 (57%)	1 (11%)		12 (44%)		0.068
5-year survival (n, %)	6 (38%)	1 (11%)		3 (14%)		0.135
Alternative/differential diagnoses		Exacerbation of interstitial pneumonia (4)		Bacterial pneumonia (8)		
		Neoplasic pleuropneumonia (1)		Cardiogenic oedema (4)		
				Exacerbation of interstitial pneumonia (3)		
		Organized pneumonia/Exacerbation of interstitial pneumonia (1)		Viral pneumonia (2)		
				Hypercapnic respiratory failure (2)		
		Bacterial pneumonia (1)		Sepsis due to urinary tract infection (2)		
				Foreign body aspiration (1)		
		Sepsis due to upper urinary tract infection (1)		Vasculitis (1)		
				Tuberculosis (1)		
		Lung transplant rejection (1)		Drug induced pneumonia (1)		
				Invasive aspergillosis (1)		
				ARDS of unknown origin (1)		

Missing data : ◊: with 1 missing data, ⊕: with 16 missing data, ♣: with 2 missing data, ♠: with 3 missing data, ♥: with 13 missing data, ♦: with 8 missing data, •: with 9 missing data, α: with 13 missing data, β: with 34 missing data, *: Predominantly perihilar and bilateral diffuse ground-glass opacities on CT (52/59) or similar on X-ray (55/59)

*PCR* Polymerase Chain Reaction, *PJP* = *Pneumocystis jirovecii* Pneumonia, *Ct* Cycle Threshold, *BDG* β-D-glucan, *ICU* Intensive Care Unit, *BMI* Body Mass Index, *LDH* Lactate Dehydrogenase, *PCT* Procalcitonin, *CRP* C-Reactive Protein, *SAPS II* Simplified Acute Physiology Score II, *CCI* = Charlson Comorbidity Index, *SOFA* Sequential Organ Failure Assessment, *SOT* Solid Organ Transplant, *Ct scan* Computed Tomography scan, *GGO* Ground Glass opacities

**Fig. 1:**
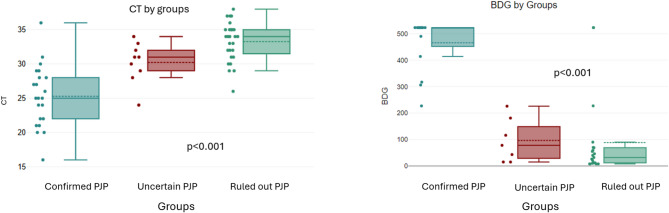
Cycles threshold values and BDG assay comparison between groups.* PJP  Pneumocystis jirovecii* Pneumonia, *Ct* Cycle threshold, *BDG* β-D-glucan

For biological parameters, there were no significant differences between confirmed and ruled out PJP patients except for median rt-PCR Ct value (median (Q1-Q3): 25 [22–28] versus 34 [32–35], *p* < 0.001), and BDG levels (median (Q1-Q3): 523 (452–523) versus 32 (11.8–69) pg/mL, *p* < 0.001). Despite the significant differences between groups, there was an overlap in Ct values and BDG levels (Fig. 1).

Only eight patients were diagnosed with proven PJP due to a positive direct examination of BAL MGG (35% of the confirmed PJP group and 14% of the positive rt-PCR group).

Among the three “ruled-out PJP” patients with BDG > 200 pg/ml, one had a diagnosis of pulmonary aspergillosis, and two had septic shock due to bacterial infections, including one with bacteremia. The distribution of BDG levels across the three groups and according to rt-PCR cycles are shown in Supplemental Fig. 2.

Nine patients (15% of the positive rt-PCR group) could not be categorized, mostly due to a high ICU mortality rate (78%), which prevented a proper evaluation of the treatment response. The description of the uncertain group is detailed in Supplemental Table 2.

Three patients had long-term prophylactic treatment against PJP, either cotrimoxazole or pentamidine; one had proven PJP and two were categorized as having an uncertain PJP. Only one patient with a PJ rt-PCR Ct value of 34 and a negative BDG assay, who was included in the ruled-out PJP group and who did not receive prophylaxis, developed PJP nearly a year after ICU admission and was not admitted to ICU at that time.

### ROC curves and best Ct threshold to categorize patients with a positive PJ rt-PCR test

In the primary analysis, the optimal PJ rt-PCR Ct threshold to categorize patients was < 30 with an AUC of 0. 931 (95% CI [0.850-1]), a sensitivity of 86%, and a specificity of 89%, as shown in Supplemental Fig. 3A. ROC curve details of the primary analysis are found in Supplemental Table 3. The BDG assay was also discriminative, with a ROC AUC of 0.933 (95% CI [0.843-1]) and an optimal threshold at 227 pg/ml.

In the secondary analysis, when adding the uncertain group to the confirmed group, the best PJ rt-PCR Ct threshold for categorized patients was 32, with an AUC of 0.888 (95% CI [0.804–0.972]), a sensitivity of 87% and a specificity of 74%. Still in the secondary analysis, when adding the uncertain group to the ruled-out group, the best PJ rt-PCR Ct value for categorized patients was 30, with an AUC of 0.907 (95% CI [0.818–0.996]), a sensitivity of 86% and a specificity of 83% (Supplemental Fig. 3, B1 and B2).

## Discussion

To the best of our knowledge, this research on the diagnostic value of PJ semi-quantitative rt-PCR performed on BAL is the first to focus exclusively on non-HIV immunocompromised and mostly non-hematologic patients admitted to the ICU with ARF.

Currently, the diagnosis of PJP relies on identifying the fungus for a proven diagnosis. Direct or indirect examination is often combined with an rt-PCR test on BAL samples. However, an rt-PCR may be positive even when direct or indirect examination is negative, raising clinical suspicion. Recent guidelines [27] have introduced the relevance of “probable PJP” criteria based on quantitative rt-PCR for non-HIV patients, which many authors use. However, a recent multicenter prospective cohort study of ICU patients with PJP showed that out of 158 patients treated for PJP, only 88 (56%) met the European criteria for proven or probable PJP [28]. This suggests that current classification methods might miss some PJP cases according to clinical judgment. In this regard, we excluded doubtful cases in our primary analysis and integrated treatment response for categorization, taking advantage to the retrospective nature of this research.

Comparing PCR diagnostic values across studies is hampered by the wide variety of patient populations, sample types and quality, DNA extraction methods, commercial or homemade PCR techniques, gene targets, and gold standards. A meta-analysis of 16 studies published in 2013 that included all types of PJP patients and various techniques of PCR performed in BAL samples only, showed a sensitivity and a specificity of 98.3% (91.3%-99.7%) and 91% (82.7%-95.5%) respectively [8]. Qualitative PCR was used to detect PJ; however, its specificity is lower than quantitative PCR, according to a meta-analysis that gathered all types of samples in patients with various profiles of immunosuppression [9]. Qualitative PCR can thus be used to rule out PJP diagnosis but cannot differentiate true infection from colonization [17].

In a review published in 2011 on 12 major studies mainly focusing on real time PCR run on BAL and including both HIV and non-HIV patients, the sensitivity ranged from 82% to 100%, and specificity ranged from 83% to 100%. These figures are higher than for qualitative PCR [12, 29, 30].

A recent study comparing various PCR tests (qPCR, reverse transcriptase PCR, homemade or commercial PCR) with different targets (whole nucleic acid (WNA), DNA, single or multi-copy genes) performed on the same samples at various dilutions by 16 laboratories in 8 countries showed that the quantification could differ by up to 12 cycles on the same specimen. This research also found a superiority for reverse transcriptase PCR targeting WNA (rather than DNA only) of the multi-copy gene mtSSU.

Our results regarding sensitivity and specificity, 100% and 94% respectively, and NPV of 100%, mean that a negative test rules out PJP. This is in line with the literature and recommendations [22], and supports the use of rt-PCR for the diagnosis of PJP in this specific population.

Among our non-HIV immunocompromised ICU patients with ARF who had a PJ rt-PCR performed on BAL, 12% had a positive result, consistent with previous studies in similar populations [26, 31]. Conversely, in the recent retrospective multicenter study from Giacobbe et al., 95 out of 147 (64%) immunocompromised ICU patients with positive PCR were ultimately diagnosed with PJP. However, that study included HIV and non-HIV immunocompromised patients, while our study focused solely on non-HIV compromised patients, which explains the lower true positive rate of PCR PJP [26]. A total of 417 patients were excluded, as their immunosuppressive conditions were deemed insufficient to confer a significant risk for PJP. None had a positive PJP PCR result, and no cases of PJP were diagnosed during follow-up. Including these patients would likely have lowered the pre-test probability and consequently reduced the diagnostic performance of the PCR. In a similar respect, patients with SARS-CoV-2 infection were excluded from the study due to the significant disruption of the healthcare system during the pandemic. Diagnostic strategies employed during this period were not representative of standard clinical practice, as they were influenced by the overwhelming number of patients requiring intensive care. In our view, these patients do not reflect the typical population in whom PJP is diagnosed, nor the usual diagnostic approach applied under standard clinical conditions.

Between our three groups of patients – those with confirmed diagnosis, those with excluded diagnosis and those whose diagnosis remained uncertain – we observed differences in terms of steroid exposure, radiologic patterns, and duration of symptoms, which suggest that these criteria should be used to identify patients with a higher pre-test probability of being diagnosed with PJP, in line with recent research [32]. Exposure to corticosteroids is a well-established risk factor for PJP, as they impair T-cell–mediated immunity, which plays a central role in the pathogenesis of the disease. The proportion of patients with septic shock was lower in the confirmed PJP group, consistent with prior observations in patients with HIV infection or hematologic malignancies, in whom septic shock is uncommon in PJP.

We found that median PJ PCR Ct values between patients with confirmed PJP and those with ruled-out PJP were significantly different, but with overlap in Ct values. These results are consistent with those published in non-HIV-infected patients, which vary greatly (from 22 to 33 Ct) [18, 29, 33]. The comparison of our 30-cycle threshold in non-HIV patients with other studies is delicate, but it aligns with the only prospective study [18]. In our population, no patient in the confirmed PJP group had a Ct threshold above 36, suggesting this threshold could help to exclude the diagnosis.

Serum BDG assays were significantly different between confirmed and ruled-out PJP patients in our study, despite some overlap. BDG testing is less sensitive in non-HIV patients than in HIV patients (86% versus 94%) [34]. The sensitivity of BDG testing can also be hampered by prior cotrimoxazole therapy [34].

Positive BDG results could be related to other fungal diseases or be falsely positive [34]. A study using the Fungitell kit found BDG levels above 80 pg/mL in 78% of ICU patients with sepsis of unknown origin, but only 18% had levels above 250 pq/mL [35].

**Fig. 2:**
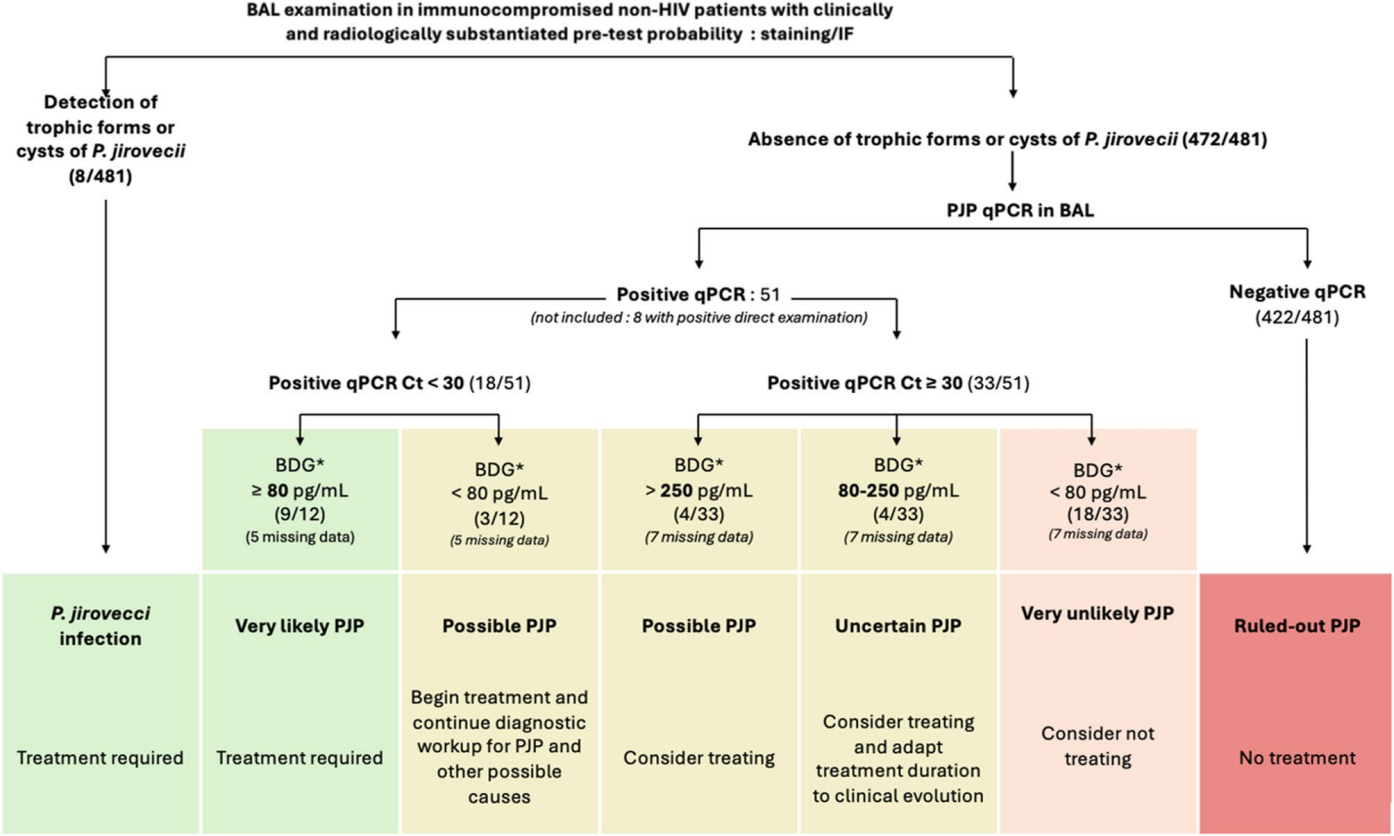
Suggested diagnostic approach for PJP in immunocompromised non-HIV ICU patients with a clinically and radiologically substantiated pre-test probability. Adapted from ECIL recommendations to non-HIV immunocompromised ICU patients (Alanio et al.); *BDG assay Fungitell to be adapted if the Wako technique is used.* PJP* *Pneumocystis jirovecii* Pneumonia,* HIV*  Human Immunodeficiency Virus, *ICU* Intensive Care Unit, *ECIL* European Conference in Infections In Leukaemia, *BAL* Broncho Alveolar Lavage, *IF* Immunofluorescence, *qPCR* semi quantitative PCR, *PCR* Polymerase Chain Reaction, *BDG* β-D-glucan

Based on our results, we suggest considering the BDG assay when PJ rt-PCR is positive, as recommended by other groups [19, 24]. In our study, all PJP confirmed patients had a highly positive BDG result. Therefore, we propose a decision tree with an algorithm based on direct examination, Ct rt-PCR values ≤ 30, and BDG level >250 pg/mL in Fig. 2.

As per guidelines, treatment decision should be individualized, and a rt-PCR Ct above 30 may be used for treatment in the most severe immunocompromised non-HIV patients, although the probability of having PJP is low [36]. A negative serum BDG result may help refine the overall diagnostic strategy. In our population, when PJP PCR cycle threshold (Ct) values were below 30, no patient in the confirmed or uncertain groups had a negative BDG result. This may reflect the high sensitivity of PJP PCR performed on bronchoalveolar lavage (BAL) samples in critically ill patients. However, as PJP has been documented in various populations despite negative BDG results, such findings are insufficient to definitively rule out the diagnosis. Instead, we suggest that a negative BDG should prompt further diagnostic investigation.

Our study has several limitations.

First, different rt-PCR kits (used during 2013–2015, 2016–2019 and 2020–2023) and DNA extraction methods (used during 2013–2019 and 2020–2023) were employed at our center during the study, which may introduce bias when comparing Ct cycles and setting a Ct threshold. This means that our threshold might not be universally accurate, but the kits we used are fully validated compared to standard methods, possess a high sensitivity and always use the same target. There was no significant difference when comparing confirmed and ruled out PJP patients, between the best Ct thresholds calculated for the last two periods. Notably, the best Ct threshold was different with the PCR test ran from 2013 to 2015 but only 7 patients were included during this span, details are shown in Supplemental Table 4. Also, there was no significant difference between the best PCR thresholds before and after 2020, when the DNA extraction method changed in our hospital as shown in Supplemental Table 5. One study suggested a similar Ct cut-off of 30 to differentiate colonization from infection [37]. Additionally, we used commercial PCRs which supports generalizability.

Second, as for other research papers [25, 26], we faced the challenge of lacking a gold standard for diagnosing PJP in non-HIV immunocompromised patients, as the diagnostic strategy that relies on pathogen identification has been validated for HIV patients with higher fungus loads. In non-HIV patients, physiopathology is different, and cases of PJP have been reported with fungus loads lower than direct identification sensitivity [11]. Our approach of considering PJP as confirmed without requiring pathogen identification has been widely used in previous studies [37], as reflected in recent revised recommendations [27]. Nevertheless, this remains a limitation, as we cannot fully exclude the possibility of PJP mimickers. Indeed, since glucocorticoid therapy was frequently continued or even increased during the ICU stay, corticosteroid-responsive pulmonary diseases may have gone unrecognized. This also limits the assessment of clinical response. Of note, although no cases of trimethoprim-sulfamethoxazole–resistant PJP have been documented in our centre, the possibility of undetected resistance cannot be ruled-out. Consistently, another limitation of our study is the absence of an adjudication committee. Three patients initially classified in the ‘uncertain PJP’ group were subject to disagreement among reviewers and were subsequently reclassified into the ‘ruled-out PJP’ group based on the expertise of the most senior reviewer. However, according to our final classification, only nine patients remained in the ‘uncertain’ category, and secondary analyses were conducted, the results of which were consistent with the primary findings.

Third, in the absence of direct pathogen identification to establish the diagnosis, clinicians often rely on chest CT findings. Another limitation of our study is the lack of detailed imaging data for all patients. However, in non-HIV, non-hematologic immunocompromised patients, the interpretation of chest CT is further complicated by the presence of pre-existing chronic pulmonary lesions, which may obscure, or mimic findings typically associated with PJP. Moreover, PJP in such patients may present with a normal chest radiograph or nonspecific imaging features. Additional patterns such as consolidation, pulmonary nodules, septal thickening, and acute respiratory distress syndrome (ARDS) have also been described [27, 38].

Fourth, reviewers had access to BDG values when classifying patients, and clinicians involved in patient care may have accessed Ct values at the time of diagnosis, potentially introducing bias.

Fifth, our study is retrospective, single-center, and suffers from a low number of confirmed PJP, so further confirmation in prospective, multicenter studies is needed. Additionally, patients with hematologic disorders were underrepresented, and our findings should be validated in other cohorts with different case mixes. PJP pathophysiology differs in HIV-positive patients, who typically show higher fungal burden and weaker inflammation. Hematologic conditions are also heterogeneous; for instance, PJP in chronic lymphocytic leukemia may resemble HIV-associated forms [39]. In non-HIV, non-hematologic patients, PJP likely presents with variable mechanisms due to differing levels of immunosuppression and inflammatory response.

Sixth, some patients with a negative PJP PCR received a few days of curative therapy before transitioning to prophylactic treatment, and we cannot exclude the possibility that this may have been sufficient to resolve a mild form of PJP.

## Conclusion

Our findings support that semi-quantitative PJ rt-PCR tests can accurately diagnose PJP in non-HIV and non-hematologic immunocompromised ICU patients with ARF, and that the number of cycles is a relevant surrogate marker for assessing DNA quantity to differentiate infection from colonization. In our study, the optimal Ct threshold for diagnosis was 30 cycles, and a negative PCR result (> 40 cycles) ruled out the diagnosis. In cases of a positive rt-PCR with a Ct value above 30 cycles, a negative serum BDG supports challenging the diagnosis.

## Supplementary Information

Supplementary material 1.

## Data Availability

The datasets used and/or analysed during the current study are available from the corresponding author on reasonable request.
