# Supplementary material for: Significance of positive semi-quantitative PCR tests on bronchoalveolar lavage for Pneumocystis jirovecii pneumonia in HIV-negative immunocompromised ICU patients with acute respiratory failure
Source: Ann Intensive Care. 2025 Oct 27;15:173. doi: 10.1186/s13613-025-01568-3 (PMC12554852; doi:10.1186/s13613-025-01568-3)
Supplement: Supplementary file 1 — Supplementary material 1. [file 13613_2025_1568_MOESM1_ESM.docx]

**Supplemental figure 1: flow chart**


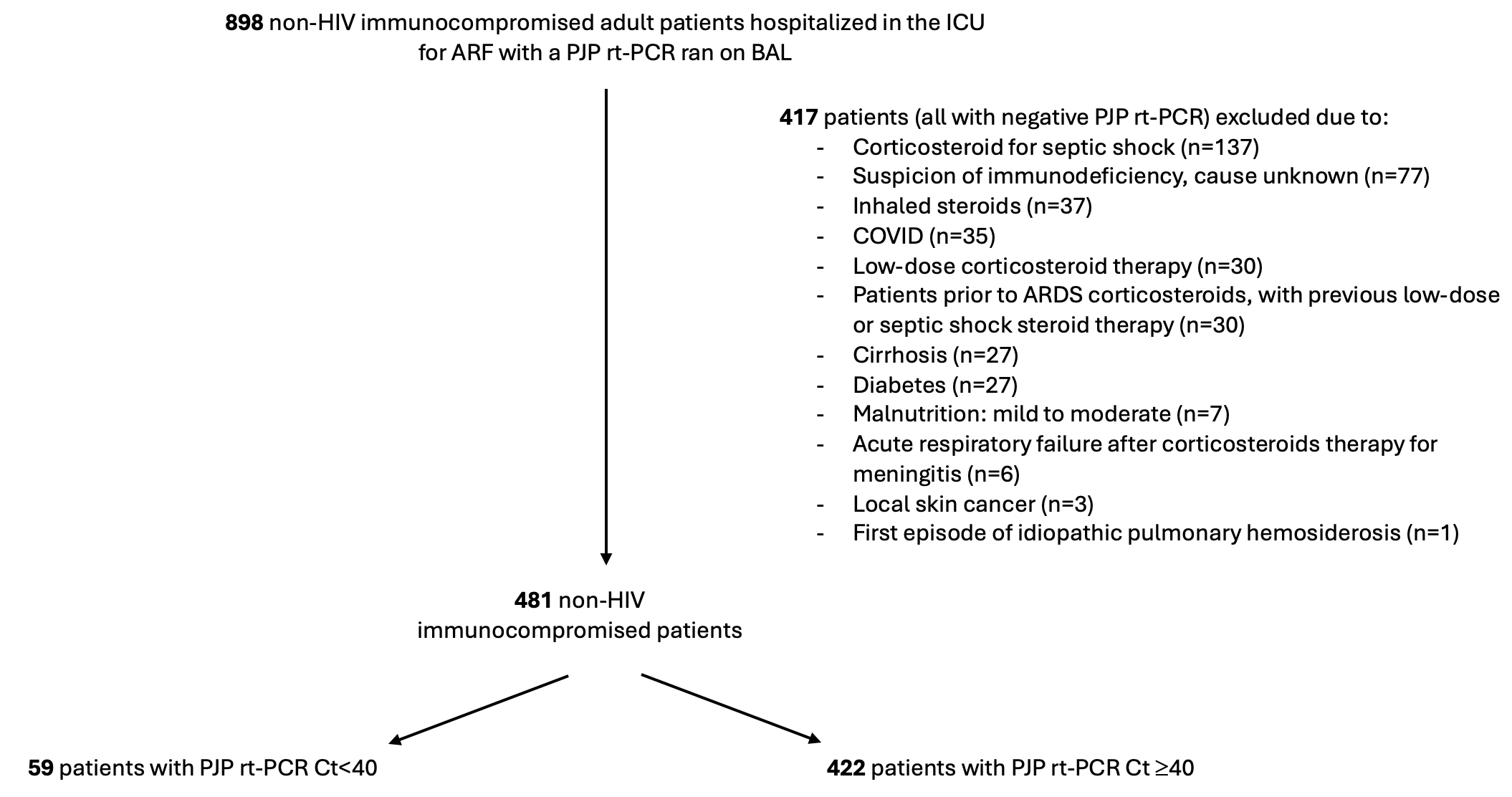


Abbreviations: HIV = Human Immunodeficiency Virus, ICU = Intensive Care Unit, ARF = Acute Respiratory Failure, PJP = *Pneumocystis jirovecii* Pneumonia, rt-PCR = Real-Time Polymerase Chain Reaction, BAL = Broncho Alveolar Lavage, Ct = Cycle Threshold.**Supplemental table 1: comparison between positive PCR and negative PCR groups**

| **Population characteristics** | **Positive PCR, n=59** | **Negative PCR, n=422** | **p values** |
| --- | --- | --- | --- |
| **Age in years (median/Q1-Q3)** | 65 (59-70) | 61 (50-68) | 0.003 |
| **Length of Stay in days (median/Q1-Q3)** | 9 (4-18) | 10 (5-22) | 0.259 |
| **Intensive Care Unit mortality (n, %)** | 38 (64%) | 112 (27%) | <.001 |
| **Saps II at admission (median /Q1-Q3)** | 36 (27-36) | 33 (13-54) | 0.026 |
| **Solid organ transplant recipient (n, %)** | 12 (20%) | 189 (45%) | <.001 |
| **Auto immune disease (n, %)** | 28 (47%) | 101 (24%) | <.001 |
| **Systemic glucocorticoid therapy before PJP suspicion (n, %)** | 41 (69%) | 262 (62%) | 0.087 |
| **Immunomodulatory therapy (n, %)** | 21 (36%) | 50 (12%) | 0.003 |
| **Hematologic malignancies (n, %)** | 2 (3%) | 39 (9%) | 0.467 |
| **Solid tumor (n, %)** | 10 (17%) | 81 (19%) | 0.68 |
| **Systemic cancer treatments (n, %)** | 10 (17%) | 43 (10%) | 0.121 |

Abbreviations: PCR = Polymerase Chain Reaction, SAPS II = Simplified Acute Physiology Score II, PJP=*Pneumocystis jirovecii* Pneumonia

**Supplemental figure 2: distribution of BDG assay across the three groups and according to PCR cycles**


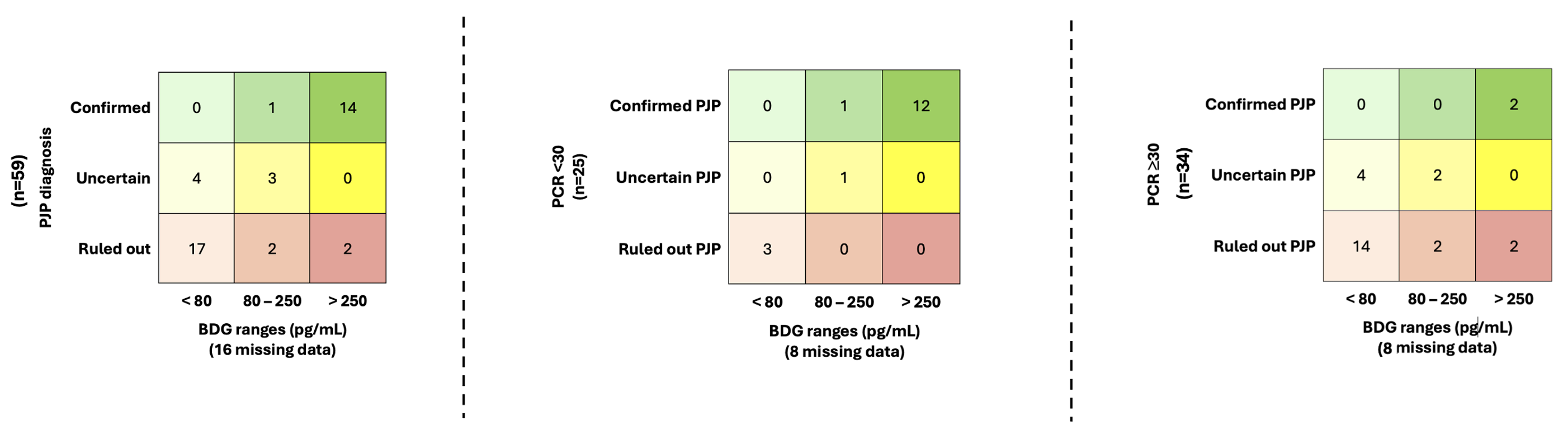


Abbreviations: BDG = β-D-glucan, PCR = Polymerase Chain Reaction, PJP = *Pneumocystis jirovecii* Pneumonia.

**Supplemental table 2: data concerning the uncertain group part 1**:

|  | **PCR CT** | **Sex** | **Age** | **LOS** | **SAPS II** | **CCI** | **SOFA** | **Immunosuppressive condition** | **Delay between onset and ICU admission** | **Xray/CT scan pattern** | **Retained / alternative diagnosis** | **Antibiotics** | **Duration of antibiotic therapy** | **Steroids during antibiotic therapy** |
| --- | --- | --- | --- | --- | --- | --- | --- | --- | --- | --- | --- | --- | --- | --- |
| **1** | 28 | F | 52 | 18 | 73 | 1 | 10 | SOTR, steroids | 0 | GG0 + Condensation | Sepsis shock (urinary tract infection) | Meropenem, quinolone | 14 | - |
| **2** | 24 | M | 64 | 8 | 77 | 8 | 7 | Solid tumor, immunosuppressive therapy, recent systemic cancer treatments | 13 | GGO + condensation + pleural effusion | Neoplasic pleuropneumonia | Piperacillin-tazobactam, amikacin | 4 | Steroids |
| **3** | 32 | M | 70 | 6 | 31 | 4 | 10 | Autoimmune disease, steroids | 7 | GGO + condensation | Acutisation of interstitial lung pneumonia | Piperacillin-tazobactam | 7 | Steroids |
| **4** | 33 | M | 64 | 12 | 46 | 4 | 3 | Chronic lymphocytic leukemia, allograft | 7 | GGO | Bacterial pneumonia | Cefepime | 7 | Steroids |
| **5** | 29 | M | 76 | 11 | 22 | 10 | 2 | Solid tumor, steroids, immunosuppressive therapy, recent systemic cancer treatments | 32 | GGO + pleural effusion | ARDS of unknown origin | Piperacillin-tazobactam + quinolone + acyclovir | Unknown | Steroids |
| **6** | 30 | F | 69 | 9 | 12 | 3 | 2 | Autoimmune disease, steroids, immunosuppressive therapy | 1 | GGO | Acutisation of interstitial lung pneumonia | Cefazolin | 7 | Steroids |
| **7** | 31 | F | 41 | 20 | 22 | 1 | 2 | SOTR, steroid | 8 | GGO | Rejection of lung transplant | Tazocillin | 6 | Steroids |
| **8** | 34 | M | 58 | 6 | 36 | 2 | 2 | Autoimmune disease, steroids | 10 | GGO | Acutisation of interstitial lung pneumonia | Amoxicillin + clavulanic acid | 8 | Steroids |
| **9** | 31 | F | 65 | 8 | 42 | 5 | 4 | Autoimmune disease, steroids, immunosuppressive therapy | 6 | GGO | Acutisation of interstitial lung pneumonia / Invasive aspergillosis and sepsis shock | Meropenem | 7 | Steroids |

**Supplemental table 2: data concerning the uncertain group part 2**:

|  | **PJP treatment** | **Duration of PJP treatment** | **ICU survival** | **Leucocytes** | **Lymphocytes** | **PNN** | **Serum LDH** | **PCT** | **CRP** | **Serum BDG** | **Possible etiology for serum BDG** |
| --- | --- | --- | --- | --- | --- | --- | --- | --- | --- | --- | --- |
| **1** | Cotrimoxazole | 21 | Yes | 16 | 1.1 | - | 451 | - | - | - | - |
| **2** | Cotrimoxazole | 9 | No | 14 | - | 13 | 1577 | - | - | - | - |
| **3** | Cotrimoxazole | 2 | No | 11 | 0.5 | 10 | 443 | 1.89 | 270 | 15 | - |
| **4** | Cotrimoxazole | 6 | No | 11 | 2.7 | 7 | 299 | 0.38 | 139 | 43 | - |
| **5** | Cotrimoxazole | 11 | No | 19 | 1.4 | 16 | 293 | 0.28 | 160 | 226 | - |
| **6** | Cotrimoxazole | 21 | Yes | 15 | 0.1 | 13 | 564 | 0.17 | 177 | 15 | - |
| **7** | Cotrimoxazole | 16 | No | 5 | 0.6 | 4 | 603 | 0.14 | - | 181 | Aspergillosis |
| **8** | Cotrimoxazole | 9 | No | 18 | 1 | 17 | 597 | 0.25 | 298 | 78 | - |
| **9** | Cotrimoxazole | 6 | No | 5 | 0.7 | 4 | 755 | 1.38 | - | 116 | Aspergillosis |

Abbreviations: PCR = Polymerase Chain Reaction, Ct = Cycle Threshold, LOS = Length Of Stay, SAPS 2 = Simplified Acute Physiology Score II, CCI = Charlon comorbidity Index, SOFA = Sequential Organ Failure Assessment, ICU = Intensive Care Unit, CT scan = Computed Tomography scan, SOTR = Solid Organ Transplant Recipient, GGO = Ground Glass Opacities, PNN = Poly Nuclear Neutrophils, LDH = Lactate Dehydrogenase, PCT = Procalcitonin , CRP = C-Reactive Protein, BDG = β-D-glucan.

**Supplemental figure 3: ROC curves**

- A - primary analysis: confirmed PJP group vs ruled-out PJP group,
- B - secondary analysis: B1- analysis with the uncertain group added to the confirmed group, B2 - secondary analysis with the uncertain group added to the ruled-out group


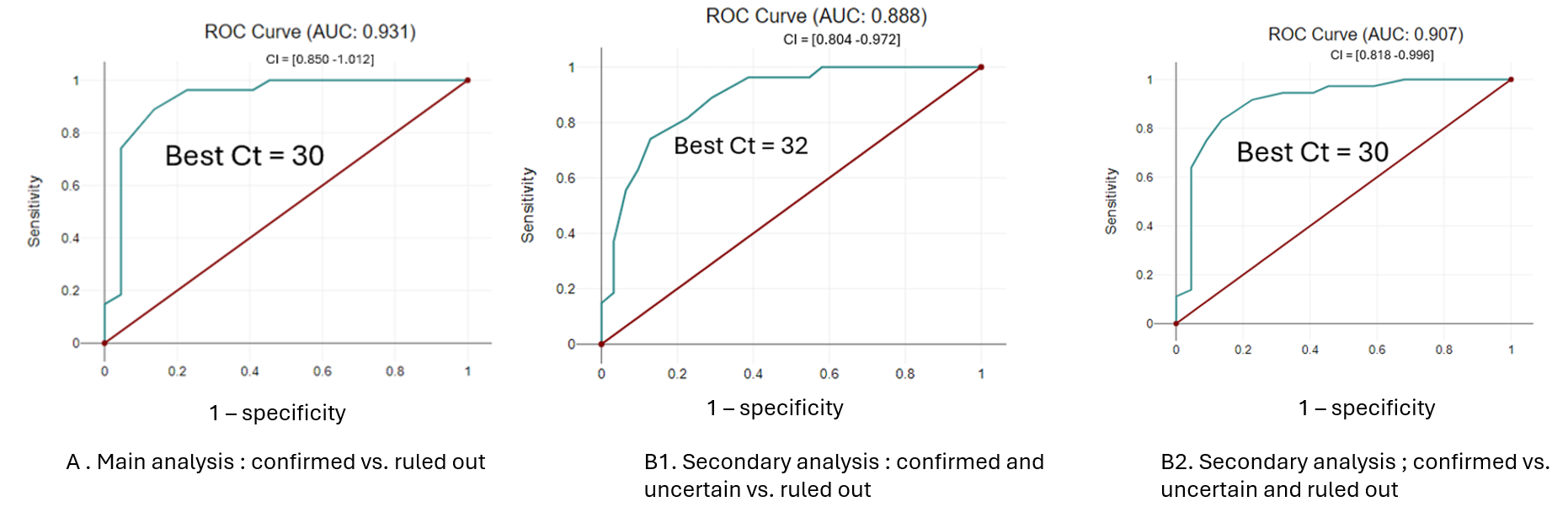


Abbreviations: ROC = Receiver Operating Characteristic, PJP = *Pneumocystis jirovecii* pneumonia, AUC = Area Under Curve, CI = Confidence Intervale, Ct = Cycle Threshold.

**Supplement table 3 : ROC details of primary analysis** (test is positive if CT < threshold value)

| **CT** | **Sensitivity** | **Lower bound (95%)** | **Upper bound (95%)** | **Specificity** | **Lower bound (95%)** | **Upper bound (95%)** | **PPV** | **NPV** | **Prevalence** | **Youden** |
| --- | --- | --- | --- | --- | --- | --- | --- | --- | --- | --- |
| <16 | 0,0% | 0,0% | 17,9% | 100% | 84,9% | 100% |  | 55,1% | 0% | 0 |
| <20 | 4,5% | 0,0% | 23,8% | 100% | 84,9% | 100% | 100% | 56,3% | 2% | 0,05 |
| <21 | 13,6% | 4,1% | 34,4% | 100% | 84,9% | 100% | 100% | 58,7% | 6% | 0,14 |
| <22 | 22,7% | 9,9% | 44,0% | 100% | 84,9% | 100% | 100% | 61,4% | 10% | 0,23 |
| <24 | 31,8% | 16,3% | 52,9% | 100% | 84,9% | 100% | 100% | 64,3% | 14% | 0,32 |
| <25 | 40,9% | 23,3% | 61,3% | 100% | 84,9% | 100% | 100% | 67,5% | 18% | 0,41 |
| <26 | 54,5% | 34,7% | 73,0% | 100% | 84,9% | 100% | 100% | 73,0% | 24% | 0,55 |
| <27 | 59,1% | 38,7% | 76,7% | 96,3% | 79,9% | 100% | 92,9% | 74,3% | 27% | 0,55 |
| <28 | 68,2% | 47,1% | 83,7% | 96,3% | 79,9% | 100% | 93,8% | 78,8% | 33% | 0,64 |
| <29 | 77,3% | 56,0% | 90,1% | 96,3% | 79,9% | 100% | 94,4% | 83,9% | 35% | 0,74 |
| <30 | 86,4% | 65,6% | 95,9% | 88,9% | 70,9% | 96,8% | 86,4% | 88,9% | 39% | 0,75 |
| <31 | 90,9% | 70,7% | 98,5% | 81,5% | 62,7% | 92,1% | 80,0% | 91,7% | 41% | 0,72 |
| <32 | 95,5% | 76,2% | 100% | 74,1% | 55,0% | 86,9% | 75,0% | 95,2% | 41% | 0,70 |
| <33 | 95,5% | 76,2% | 100% | 63,0% | 44,1% | 78,4% | 67,7% | 94,4% | 41% | 0,58 |
| <34 | 95,5% | 76,2% | 100% | 55,6% | 37,3% | 72,4% | 63,6% | 93,8% | 41% | 0,51 |
| <35 | 95,5% | 76,2% | 100% | 37,0% | 21,6% | 55,9% | 55,3% | 90,9% | 41% | 0,32 |
| <36 | 95,5% | 76,2% | 100% | 18,5% | 7,9% | 37,3% | 48,8% | 83,3% | 43% | 0,14 |
| <37 | 100% | 82,1% | 100% | 14,8% | 5,4% | 33,3% | 48,9% | 100% | 45% | 0,15 |
| <38 | 100% | 82,1% | 100% | 3,7% | 0,0% | 20,1% | 45,8% | 100% | 45% | 0,04 |

Abbreviations: ROC = Receiver Operating Characteristic, CT = Cycle Threshold, PPV = Positive Predictive Value, NPV = Negative Predictive Value.

**Supplement table 4: best CT thresholds according to PCR kits**

|  | **Best Ct Threshold** | **AUC** | **Standard error** | **Lower bound (95%)** | **Upper bound (95%)** | **Sensitivity** | **Lower bound (95%)** | **Upper bound (95%)** | **Specificity** | **Lower bound (95%)** | **Upper bound (95%)** | **PPV** | **Lower bound (95%)** | **Upper bound (95%)** | **NPV** | **Lower bound (95%)** | **Upper bound (95%)** | **LR+** | **LR-** | **Accuracy** |
| --- | --- | --- | --- | --- | --- | --- | --- | --- | --- | --- | --- | --- | --- | --- | --- | --- | --- | --- | --- | --- |
| 2013-2015 | < 35 | **0,83** | 0,00 | 83% | 83% | 83% | 42% | 98% | 100% | 17% | 100% | 1 | 51% | 100% | 0,50 | 10% | 90% | +Inf | 0,17 | 0,86 |
| 2016-2019 | < 31 | **0,96** | 0,03 | 90% | 100% | 100% | 65% | 100% | 85% | 56% | 97% | 0,82 | 51% | 96% | 1 | 69% | 100% | 6,50 | 0,00 | 0,91 |
| 2020-2023 | < 29 | **0,94** | 0,05 | 85% | 100% | 71% | 35% | 92% | 100% | 73% | 100% | 1 | 51% | 100% | 0,87 | 61% | 97% | +Inf | 0,29 | 0,90 |

Abbreviations: CT = Cycle Threshold, PCR = Polymerase Chain Reaction, CT = Cycle Threshold, AUC = Area Under Curve, PPV = Positive Predictive Value, NPV = Negative Predictive Value, LR+/- = Likelihood ratio +/-, Inf = Infinite.

**Supplement table 5: best CT thresholds according to DNA extraction method**

|  | **Best Ct Threshold** | **AUC** | **Standard error** | **Lower bound (95%)** | **Upper bound (95%)** | **Sensitivity** | **Lower bound (95%)** | **Upper bound (95%)** | **Specificity** | **Lower bound (95%)** | **Upper bound (95%)** | **PPV** | **Lower bound (95%)** | **Upper bound (95%)** | **NPV** | **Lower bound (95%)** | **Upper bound (95%)** | **LR+** | **LR-** | **Accuracy** |
| --- | --- | --- | --- | --- | --- | --- | --- | --- | --- | --- | --- | --- | --- | --- | --- | --- | --- | --- | --- | --- |
| 2013-2019 | < 31 | 0,91 | 0,06 | 79% | 100% | 93% | 68% | 100% | 86% | 59% | 97% | 0,88 | 62% | 98% | 0,92 | 64% | 100% | 6,53 | 0,08 | 0,90 |
| 2020-2023 | < 29 | 0,94 | 0,05 | 85% | 100% | 71% | 35% | 92% | 100% | 73% | 100% | 1 | 51% | 100% | 0,87 | 61% | 97% | +Inf | 0,29 | 0,90 |

Abbreviations: CT = Cycle Threshold, DNA = Deoxyribonucleic Acid, CT = Cycle Threshold, AUC = Area Under Curve, PPV = Positive Predictive Value, NPV = Negative Predictive Value, LR+/- = Likelihood ratio +/-, Inf = Infinite.
